# A Case of Pulmonary Hemorrhage, Supratherapeutic International Normalized Ratio (INR), and Anti-neutrophil Cytoplasmic Antibody (ANCA)-Associated Vasculitis: Unmasking a Potential Link to Tirzepatide

**DOI:** 10.7759/cureus.80539

**Published:** 2025-03-13

**Authors:** Husam Shakour, Reem Mumtaz, Edwin Feghali, Abdel-Ghanie Abu-Samra

**Affiliations:** 1 Internal Medicine, University of Kansas School of Medicine - Wichita, Wichita, USA; 2 Pulmonology and Critical Care, University of Kansas School of Medicine - Wichita, Wichita, USA

**Keywords:** anca associated vasculitis, medication interaction, pulmonary hemorrhage, tirzepatide, warfarin

## Abstract

Glucagon-like peptide-1 receptor agonists (GLP-1RAs), such as tirzepatide (Mounjaro), are widely used for type 2 diabetes and obesity, but their effects on drug metabolism and immune regulation remain areas of concern. We report a 64-year-old male with diabetes and chronic deep vein thrombosis (DVT) on warfarin who developed pulmonary hemorrhage, acute kidney injury (AKI), and markedly elevated cytoplasmic anti-neutrophil cytoplasmic antibody (c-ANCA) levels shortly after increasing his tirzepatide dose. His international normalized ratio (INR) was supratherapeutic at 8.7, prompting warfarin discontinuation. Despite INR normalization, he developed diffuse alveolar hemorrhage (DAH) and worsening renal function. Workup revealed ANCA-associated vasculitis, though confirmation by renal biopsy was pending at the time of the patient’s expiration. He required corticosteroids, plasmapheresis, rituximab, and hemodialysis. This case highlights potential pharmacokinetic interactions between tirzepatide and warfarin, as well as a possible role for tirzepatide in triggering autoimmunity. While definitive causality remains unclear, clinicians should closely monitor INR levels in patients on GLP-1RAs and warfarin and be vigilant for autoimmune complications in those presenting with pulmonary-renal syndromes. Further research is needed to explore the immunomodulatory effects of tirzepatide.

## Introduction

The emergence of glucagon-like peptide-1 receptor agonists (GLP-1RAs), including tirzepatide (Mounjaro), has revolutionized the management of type 2 diabetes mellitus and obesity. These agents enhance glycemic control by stimulating insulin secretion and delaying gastric emptying. However, their impact on the absorption and metabolism of co-administered medications, particularly those with narrow therapeutic indices, such as warfarin, necessitates closer observation.

GLP-1RAs delay gastric emptying, which may prolong warfarin absorption and contribute to unpredictable fluctuations in the international normalized ratio (INR) [1,2]. Additionally, warfarin is highly protein-bound, and conditions such as hypoalbuminemia can increase its free active fraction, heightening anticoagulation effects [3,4]. Although studies have documented INR alterations in patients using GLP-1RAs, severe bleeding complications remain uncommon [1,2].

Moreover, weight loss associated with GLP-1RAs can contribute to malnutrition, further reducing serum albumin levels and amplifying warfarin’s anticoagulant effect [5]. These pharmacokinetic considerations highlight the importance of vigilant INR monitoring in patients receiving both medications.

We present the case of a 64-year-old male who developed pulmonary hemorrhage, acute kidney injury (AKI), and markedly elevated cytoplasmic anti-neutrophil cytoplasmic antibody (c-ANCA) levels following an escalation in his tirzepatide dose. While the interplay between GLP-1RAs and warfarin poses inherent risks, this case raises an additional concern: the potential for tirzepatide to contribute to the development of ANCA-associated vasculitis.

Beyond pharmacokinetic interactions, this case was further complicated by diffuse alveolar hemorrhage (DAH), and AKI in the setting of elevated c-ANCA, suggesting a possible diagnosis of ANCA-associated vasculitis. This autoimmune condition commonly presents as pulmonary-renal syndrome, manifesting with hemoptysis, glomerulonephritis, and systemic inflammation [6].

The severity of pulmonary hemorrhage in this patient suggested an underlying pathology beyond anticoagulation alone. The markedly elevated c-ANCA and renal biopsy findings supported the presence of ANCA-associated vasculitis, potentially unmasked by warfarin use. The temporal relationship between the patient’s initiation of tirzepatide five months before presentation, his recent dose escalation, and the onset of vasculitis raises the possibility that tirzepatide itself may have contributed to triggering the autoimmune response.

## Case presentation

A 64-year-old male with a past medical history significant for hypertension, treated with amlodipine; type 2 diabetes, treated with metformin and tirzepatide due to concomitant obesity; and a history of chronic bilateral deep vein thrombosis (DVT) on warfarin, presented with hemoptysis, hematuria, and worsening renal function. Two days prior to admission, he had increased his weekly tirzepatide dose from 7.5 mg to 10 mg, which he had been on for five months. A routine lab check revealed an INR of 8, prompting his physician to discontinue warfarin and send him to the hospital.

On presentation, the patient reported hemoptysis (approximately one tablespoon), hematuria, and a right subconjunctival hemorrhage. His vital signs were notable for sinus tachycardia (heart rate, or HR: 106 bpm) and stable oxygenation (SpO_2_ 95% on room air). Physical examination revealed diffuse rhonchi on pulmonary auscultation. Laboratory findings included hemoglobin of 10.5 g/dL, platelet count of 486 × 10^9/L, serum creatinine of 1.71 mg/dL (baseline 1.17), and an INR of 8.7. Serum albumin was low at 2.8 g/dL (Table 1).

**Table 1:** Laboratory results for patient's hospital stay INR, International normalized ratio; c-ANCA, Cytoplasmic anti-neutrophil cytoplasmic antibody

Test	Result	Reference Range/Units
Hemoglobin	10.5	13.5 - 17.5 g/dL
Platelet count	486	150 - 400 × 10⁹/L
Serum creatinine	1.17 to 1.71 to 5.2	0.7 - 1.3 mg/dL
INR	8.7	0.8 - 1.2 (normal) or 2.0 - 3.0 (therapeutic on warfarin)
Serum albumin	2.8	3.5 - 5.0 g/dL
c-ANCA	2213	<20 U/mL

**Figure 1:**
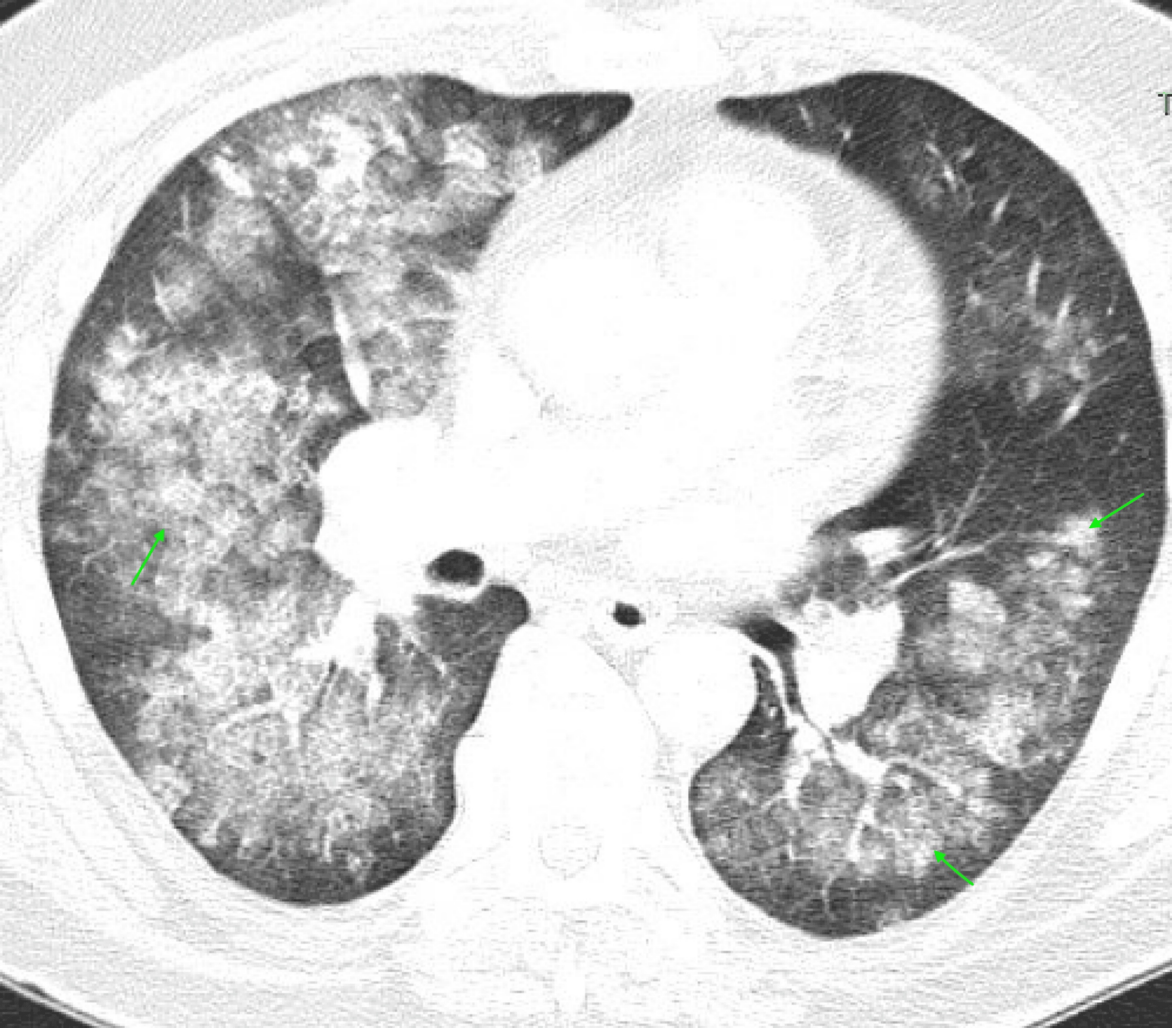
A computed tomography (CT) angiography of the chest Diffuse bilateral perihilar infiltrates with patchy ground-glass opacities (arrows), consistent with pulmonary hemorrhage.

**Figure 2:**
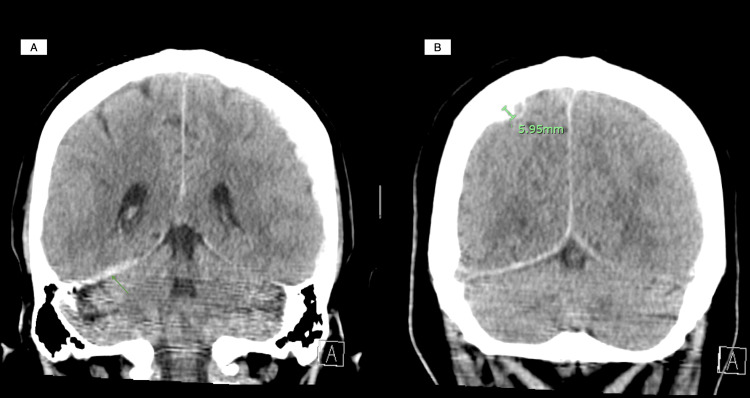
A non-contrast computed tomography (CT) of the brain (A) Acute trace subdural blood along the posterior falx and right tentorium; (B) Acute right convexity subdural hematoma measuring 5.95 mm.

A computed tomography (CT) angiography of the chest revealed diffuse bilateral perihilar infiltrates with patchy ground-glass opacities, consistent with pulmonary hemorrhage but without pulmonary embolism (Figure 1). A renal ultrasound was unremarkable, and a non-contrast CT brain identified small bilateral subdural hematomas without mass effect (Figures 2A-2B).

Management included intravenous vitamin K, which normalized INR to 1.5, and inhaled tranexamic acid for hemoptysis. However, bleeding persisted, with hemoglobin trending down to 7 g/dL. His course was further complicated by atrial fibrillation with rapid ventricular response, requiring rate control with metoprolol and amiodarone. Given his high bleeding risk, anticoagulation was held, and an inferior vena cava (IVC) filter was placed for DVT management.

Recognizing the unusual severity of DAH despite an elevated INR, autoimmune workup revealed a markedly elevated c-ANCA at 2213 U/mL, prompting a renal biopsy. Despite high-dose corticosteroids (methylprednisolone 500 mg for three days, followed by prednisone taper), renal function deteriorated, peaking at a creatinine of 5.2 mg/dL. Due to worsening uremic symptoms and altered mental status, the patient underwent hemodialysis.

His hospitalization was further complicated by status epilepticus, requiring ICU admission and intubation. Given his refractory seizures, he received multiple anti-epileptics, including levetiracetam, phenytoin, and lacosamide. Plasmapheresis (PLEX) and rituximab were initiated as part of his vasculitis management.

Unfortunately, the patient’s condition continued to deteriorate, and he ultimately passed away, preventing further follow-up on the kidney biopsy.

## Discussion

This case underscores the complex interplay between GLP-1RAs, warfarin metabolism, and autoimmune pathology. The patient’s supratherapeutic INR following a tirzepatide dose increase suggests a pharmacokinetic interaction, likely exacerbated by malnutrition and hypoalbuminemia. These factors, combined with the anticoagulant effects of warfarin, created a setting in which bleeding complications became significantly more pronounced.

Additionally, drug-induced autoimmune diseases, including vasculitis and lupus-like syndromes, have been documented for decades [7]. Given that tirzepatide was patented in 2016 [8], and its long-term effects are still being explored, its potential immunomodulatory properties warrant attention. GLP-1RAs have been shown to influence the innate immune response, particularly by affecting macrophages and key inflammatory pathways such as NF-κB [9]. Although some studies suggest that GLP-1RAs influence immune modulation, we have not found any evidence in the literature directly linking tirzepatide to the development of autoimmune conditions such as ANCA-associated vasculitis.

Several alternative causes of ANCA-associated vasculitis were considered and ruled out in this case. Infectious etiologies were excluded based on negative blood cultures and viral panels. Anti-glomerular basement membrane (anti-GBM) disease and systemic lupus erythematosus (SLE) were ruled out with negative anti-GBM and antinuclear antibodies (ANAs), respectively. Medication-induced vasculitis was also explored, but the patient was not on other known vasculitis-triggering drugs, such as hydralazine, propylthiouracil, or minocycline [7]. No underlying malignancy or hematologic disorder was identified that could explain a paraneoplastic vasculitic process.

The temporal association observed in this case - where the initiation and subsequent dose escalation of tirzepatide coincided with supratherapeutic INR levels and a new diagnosis of ANCA vasculitis - raises important questions about a possible causal link. While definitive causality cannot yet be established, this case highlights the need for further research into potential autoimmune sequelae associated with GLP-1RAs. The possibility that tirzepatide may trigger an immune-mediated response, either directly or through its effects on inflammatory pathways, should be explored in future clinical studies.

This case also emphasizes the importance of diligent monitoring when initiating or adjusting GLP-1RAs in warfarin-treated patients. Close INR surveillance, nutritional assessments, and vigilance for potential autoimmune triggers are essential to mitigate risks. While the connection between tirzepatide and vasculitis remains speculative, this case reinforces the need for continued pharmacovigilance and deeper investigation into emerging medication interactions.

## Conclusions

This case highlights the potential interaction between GLP-1RAs, warfarin metabolism, and autoimmunity. While causality remains uncertain, it underscores the need for close INR monitoring and vigilance for autoimmune complications in patients on GLP-1RAs. Further research is warranted to explore their immunomodulatory effects.

## References

[1] Anjum P, Akbashev M, Utz A, Kisala S (2024). Warfarin and GLP-1 receptor agonist interaction effects on time in therapeutic range. Blood.

[2] Maideen NMP (2019). Pharmacologically relevant drug interactions of glucagon-like peptide-1 receptor agonists. J Anal Pharm Res.

[3] Calvarysky B, Dotan I, Shepshelovich D, Leader A, Cohen TD (2024). Drug-drug interactions between glucagon-like peptide 1 receptor agonists and oral medications: a systematic review. Drug Saf.

[4] Horton JD, Bushwick BM (1999). Warfarin therapy: evolving strategies in anticoagulation. Am Fam Physician.

[5] Kawai M, Harada M, Motoike Y, Koshikawa M, Ichikawa T, Watanabe E, Ozaki Y (2019). Impact of serum albumin levels on supratherapeutic PT-INR control and bleeding risk in atrial fibrillation patients on warfarin: a prospective cohort study. Int J Cardiol Heart Vasc.

[6] Comarmond C, Cacoub P (2014). Granulomatosis with polyangiitis (Wegener): clinical aspects and treatment. Autoimmun Rev.

[7] Hogan JJ, Markowitz GS, Radhakrishnan J (2015). Drug-induced glomerular disease: immune-mediated injury. Clin J Am Soc Nephrol.

[8] (2025). Espacenet - patent search. https://worldwide.espacenet.com/publicationDetails/biblio?CC=US&NR=2016199438A1&KC=A1&FT=D&ND=&date=20160714&DB=&locale=en_EP.

[9] Chen J, Mei A, Wei Y (2022). GLP-1 receptor agonist as a modulator of innate immunity. Front Immunol.

